# Meeting of the American Medical Association

**Published:** 1871-03

**Authors:** 


### Editorial.

### Meeting of the American Medical Association.—Fare to San Francisco and Back, Time and Expenses of the Journey, &c., &c.

The forth-coming meeting of the American Medical Association is exciting great interest in the profession; and from the present inquiries upon the subject, we infer that the States will furnish a large delegation, notwithstanding the great distance and conseqnent length of time which will be necessarily occupied. The importance of the topics upon which committees are to report. the novelty of a journey to the Pacific Coast, and a general desire to advance the interests of the Association, will combine to increase the number who will attend; and we bespeak one of the most successful meetings of the Association.

It may be, as it was last year in Washington, that some side issues will be crowded upon the attention. As the Negro question was SETTLED in Washington, so the Chimpanzee and Chinaman questions may possibly obtain final adjustment in San Francisco. However, if it shall be shown that the Orang tribe is really the great Ancestral Father of us all, ourselves having decended or rather ascended by progressive development, the profession will scarcely feel like ignoring any of the links in the great chain; but, in California, will admit to the rights and privileges of the Association, all educated regular physicians, either as delegates or members by invitation, not hesitating on account of race, color or origin.

There is evidently in store for the profession a grand entertainment; and those who may have time enough, and money enough, will do well to accept the inviattion. We have received invitation, in addition to the general invitation which all physicians receive, to also attend and join the Association of Editors of Medical Journals, which is to hold its annual meeting at San Francisco. The desire to promote the objects of the Association is an additional incentive to us to attend; but, alas! the profits of Medical Journalism are not received in such currency as will generally purchase berths in Pullman Sleeping Cars for the Pacific.

We can imagine the conductor's astonishment on being offered in payment some of our "scrip." It would read thus:

BUFFALO, March 25th, 1871.

EDITOR OF THE BUFFALO MEDICAL AND SURGICAL JOURNAL:

To BAKER JONES & COMPANY, DR.

To One Thousand Journals, March No., . . . . . . . . . . . $200.00.

Received Payment,

No name after received payment, and the following: "P. S.—Please pay bearer the above bill without delay, and greatly oblige, yours, &c., Baker, Jones & Co." Conductor looks over the paper, seems bewildered and unable to make out the case. Finally he says, "What *is* this?" "*Our* currency, sir!" Conductor passes back a little to get a better view of the "check," and a better view of the Editor and his company. The appearance is all very respectable, the company is unexceptionable, and he is hardly ready to put a quiet, well-behaved gentleman off the cars until he *knows* that there is something very wrong about it, so he inquires "Is this good for its face?" A traveling companion, who knows the Editor, now interposes an endorsement, assuring him that the "scrip" is as good as gold, and will be paid promptly when—the subscribers to the Journal pay their dues (in advance)—the "in advance" being said in an under tone, so that the conductor finally accepts the "scrip," and says he has no doubt it is all right, and that Mr. Pullman will be glad to send all the Medical Editors upon the same terms. Upon such terms no reduction of fare will be expected. In furtherance, therefore, of the objects of this Association, all Editors holding similar evidences of debt are advised to offer it in payment of fare, and see if it will pass.

The permanent secretary, Dr. Atkinson, of Philadelphia, who has done more for the prosperity of the Association than any other man, has already invited us to attend the meeting. We rather guess he wants to have us make report of the proceedings. *Our* report last year was received by the profession with the heartiest demonstrations of approval. If we can pay our fare in the manner aforesaid, we pledge our readers a full and accurate report of the incidents of the journey and Proceedings of the Association.

Since writing the above, we have received the following letter from Wm. B. Atkinson, M. D., Permanent Secretary:

AMERICAN MEDICAL ASSOCIATION,
Office of Permanent Secretary, WM. B. ATKINSON, M. D.,
1400 Pine St., S. W. cor. Broad, PHILADELPHIA, March 8, 1871.

Dr. J. F. MINER:

Dear Doctor,—The fare from New York to San Francisco and back, $170. From Chicago and back, $149.

At Omaha, all must have certificate from *me* to procure tickets over the U. P. R. R.

From Omaha to San Francisco and back, $125. Tickets sold *only* to holders of *my* certificate, stating name, &c.

Am yet in treaty for better terms, but fear a failure.

Please state in your Journal, that those desiring certificates must apply early with the names of all their party.

As soon as I can say more, I will do so.

Truly, in haste,

WM. B. ATKINSON.

AMERICAN MEDICAL ASSOCICTION,
Office of Permanent Secretary, WM. B. ATKINSON,
1400 Pine St., S. W. corner Broad.

PHILADELPHIA, March 24, 1871.

ARRANGEMENTS FOR THE MEETING ON TUESDAY, MAY 2,
AT SAN FANCISCO, CAL.

Union and Central Pacific Railroad: From Omaha to San Francisco and return, $125. Tickets good for 60 days, and sold *only* to holders of certificate from Permanent Secretary. This includes the wives and families of ALL who desire to participate in this excursion. Each person must be named in the certificate.

From Harrisburg to Omaha and return, $49. From Philadelphia, $53.20. Tickets sold *only* to holders of certificate as above.

To Omaha: From Cincinnati, Louisville, Nashville, one fare for the round trip. From Washington, $59.30.

Local arrangements have been made with other roads; hence application should be made at starting for *excursion* tickets.

TIME.—From Omaha to San Francisco, nearly 4 days; To Omaha from Boston, 64 hours; New York, 62 hours; Philadelphia, 58 hours; Washington, 60 hours; Chicago, 22 hours.

MEALS.—At convenient points, and good, 75 cents to $1.00.

SLEEPING CARS.—Each double berth, Omaha to Ogden, $8; Ogden to San Francisco, $6.

Passengers will be taken by the Pacific Mail Steamship line, *via* Panama, at one-third less fare, either way. Tickets sold *only* to holders of certificates.

Those desiring certificates should apply immediately, inclosing *stamp*.

WM. B. ATKINSON.

N. B.—It is suggested that as many as possible should be at Omaha by April 26th or 27th, at the latest, reaching San Francisco the day before the meeting.

